# Approximation of a physiologically structured population model with seasonal reproduction by a stage-structured biomass model

**DOI:** 10.1007/s12080-016-0309-9

**Published:** 2016-09-01

**Authors:** Floor H. Soudijn, André M. de Roos

**Affiliations:** Institute for Biodiversity and Ecosystem Dynamics, University of Amsterdam, Amsterdam, The Netherlands

**Keywords:** Seasonal reproduction, Population size-distribution, Size-structured model, Consumer-resource, Model derivation, Population dynamics

## Abstract

Seasonal reproduction causes, due to the periodic inflow of young small individuals in the population, seasonal fluctuations in population size distributions. Seasonal reproduction furthermore implies that the energetic body condition of reproducing individuals varies over time. Through these mechanisms, seasonal reproduction likely affects population and community dynamics. While seasonal reproduction is often incorporated in population models using discrete time equations, these are not suitable for size-structured populations in which individuals grow continuously between reproductive events. Size-structured population models that consider seasonal reproduction, an explicit growing season and individual-level energetic processes exist in the form of physiologically structured population models. However, modeling large species ensembles with these models is virtually impossible. In this study, we therefore develop a simpler model framework by approximating a cohort-based size-structured population model with seasonal reproduction to a stage-structured biomass model of four ODEs. The model translates individual-level assumptions about food ingestion, bioenergetics, growth, investment in reproduction, storage of reproductive energy, and seasonal reproduction in stage-based processes at the population level. Numerical analysis of the two models shows similar values for the average biomass of juveniles, adults, and resource unless large-amplitude cycles with a single cohort dominating the population occur. The model framework can be extended by adding species or multiple juvenile and/or adult stages. This opens up possibilities to investigate population dynamics of interacting species while incorporating ontogenetic development and complex life histories in combination with seasonal reproduction.

## Introduction

Seasonal reproduction is a common mode of reproduction, especially in the temperate zone (Vila-Gispert et al. 2002; Félix et al. 2011). It is one of the most obvious causes of temporal fluctuations in populations. Host-parasite dynamics have been shown to be affected by seasonal reproduction due to seasonal variation in suitable hosts and vector abundance (White et al. 1996; Altizer et al. 2006; Grassly and Fraser 2006). Seasonal reproduction also has a profound effect on predator-prey dynamics. For example, the timing of reproduction of a predator relative to the seasonal peak in its prey availability may determine its recruitment success (Cushing 1990; Durant et al. 2007). It is even hypothesized that the seasonality of reproductive output structures trophic cascades and the extent of top-down control in tri-trophic food chains (van Leeuwen et al. 2007; Nakazawa and Doi 2012). Thus, seasonal reproduction shapes population and community dynamics.

Modeling seasonal processes has a long history in population ecology. Nicholson and Bailey (1935) first implemented seasonal reproductive pulses in a model describing parasitoid-host dynamics. In this classic study, the interaction between annual, discrete generations of parasitoids, and their insect hosts was analyzed. Classical models for single fish stock dynamics also often consider pulsed annual breeding using discrete time equations (Hilborn and Walters 1992). The underlying individual-level assumptions that result in single population models in terms of discrete equations account for competition between the individuals of a population but assume that these individuals are identical and do not change in their traits between reproductive events, for example they do not grow in body size (Geritz and Kisdi 2004; Brännström and Sumpter 2005). However in reality, animal and plant populations in nature rarely consist of a collection of identical individuals and individuals do change, in particular in body size, between reproduction events. These factors result in a dynamical complexity that cannot easily be caught in discrete-time equations, especially when dynamics are somehow nonlinear. The mix of continuous and discrete processes requires a hybrid model type, a so-called semi-discrete model (Murdoch et al. 2003; Pachepsky et al. 2008).

Recently, awareness of the importance of intra-specific variation for population and community dynamics has taken a flight (Yang and Rudolf 2010; Bolnick et al. 2011; Miller and Rudolf 2011; Kendall 2015). One of the most fundamental differences between individuals in a population stems from their ontogenetic development (Werner and Gilliam 1984). In most animal species, except for species of birds and mammals, drastic changes occur, in for example body size, over the development through life. These changes have profound consequences for energy acquisition and energy use in individuals (Peters 1983; Kooijman 2000; Persson and de Roos 2013). Including differences in body size over ontogeny and basic principles of animal metabolism (i.e., somatic growth is a food-dependent process and maintenance of the body requires energy) in population models, has been shown to strongly affect population and consumer-resource dynamics (Schröder et al. 2009; Persson and de Roos 2013; Schröder et al. 2014).

Seasonal reproduction inevitably results in fluctuations of the population size distribution over the year as seasonal reproduction represents an influx of small individuals in the reproductive season while later in the year the abundance of small individuals declines through somatic growth and mortality. In addition, energetic processes in individuals are affected by the reproductive rhythm (McBride et al. 2013). When reproduction occurs continuously throughout the year, energy can be directly invested in reproduction. However, when reproduction is concentrated during a certain period of the year, reproduction requires storage of energy (Bonnet et al. 1998; McBride et al. 2013). Seasonal variation in population size distributions may also be crucial for the transfer of energy between different trophic levels, since predation is usually size-specific (Peters 1983; Ebenman and Persson 1988). It has been shown that the time of hatching of a predator relative to the time of hatching of its prey determines the performance of predators (Rasmussen et al. 2014; Nosaka et al. 2015). Moreover, it is thought that seasonal reproduction may structure the extent of trophic control and trophic cascades in food chains (van Leeuwen et al. 2007; Nakazawa and Doi 2012). To study the potentially profound effects of seasonal reproduction on community dynamics, it is essential to take population size-structure, individual-level energetics, and size-dependent predator-prey interactions into consideration.

Size-structured consumer-resource models that consider (1) seasonal reproduction, (2) an explicit growing season, and (3) individual-level energetic processes exist in the form of physiologically or size- structured population models (PSPMs) (Persson et al. 1998; Claessen et al. 2000; van de Wolfshaar et al. 2008; van Leeuwen et al. 2013). However, modeling large numbers of interacting species using a PSPM framework becomes vastly complex. The studies with PSPMs so far describe maximally two interacting size-structured populations and analysis of larger species ensembles seems only feasible with a simpler modeling framework. To facilitate the analysis of community dynamics of multiple size-structured populations based on an energetics approach, de Roos et al. (2008) derived a stage-structured approximation to a PSPM with continuous reproduction. This so-called stage-structured biomass model has been used to analyze dynamics of several species communities (van Leeuwen et al. 2008; van de Wolfshaar et al. 2012; van Denderen and van Kooten 2013). The derivation of the stage-structured biomass model from the PSPM is based on the pseudo-steady-state assumption that the size distribution in the juvenile life-stage is stable and equal to the size distribution characteristic for the prevailing food density (de Roos et al. 2008). This assumption obviously does not hold in case reproduction is implemented as a seasonal process since seasonal reproduction automatically results in fluctuations in the population size-distribution. It is therefore questionable whether a stage-structured biomass model can approximate a PSPM with seasonal reproduction.

In this study, we derive a stage-structured biomass model that approximates a PSPM with seasonal reproduction. Both models assume that growth, investment in reproduction, and maturation are food-dependent processes that take place throughout the growing season. Reproductive energy is stored until the reproductive season. Spawning is assumed to be a set, seasonal event that is performed at the same time by all individuals in the population. We compare numerical simulations of the two models when juveniles and adults are equal competitors for the resource (ontogentic symmetry: Persson and de Roos 2013; de Roos et al. 2013) and when either juveniles or adults are superior at ingesting the resource (ontogentic asymmetry: Persson and de Roos 2013; de Roos et al. 2013). In addition, we investigate the effect of consumer body size and the number of juvenile stages of the stage-structured biomass model on the dynamics predicted by the two model approaches. Species that are small have short lifetimes, few reproductive events, and high energetic rates; species that are large live long and have many reproductive events but low energetic rates. We show that, with seasonal reproduction, average biomass densities of a PSPM are reasonably well-represented by average biomass densities of the stage-structured biomass model that we derive.

## Model formulation

The two models that we describe in this paper are based on the same individual-level assumptions regarding food ingestion and bioenergetics, and energy investment in somatic growth and reproduction. In this regard, the models follow the size-structured population models such as described earlier by de Roos et al. (2008), de Roos et al. (2013), and de Roos and Persson (2013). The individual energy budget follows a bioenergetics approach similar to the one used by Yodzis and Innes (1992), in which net-biomass production is a balance between assimilated energy and maintenance.

### Individual-level processes

Individuals are born with body size *Sb* and are assumed to mature at size *Sm*. Over their life history, growth in body size, maturation, and investment in reproduction are food-dependent processes. During their juvenile phase, individuals invest all their energy in somatic growth while as adults they invest all their energy in reproduction. While increasing in size, foraging rates and metabolic rates increase linearly with body size. In the following, these processes will therefore consistently be represented by their mass-specific rates.

Food ingestion follows a Holling type II functional response as a function of resource density *R* and the net-biomass production per unit body mass for juvenile *ν*_*J*_(*R*) and adult *ν*_*A*_(*R*) consumers is given by:

1 $$\begin{array}{@{}rcl@{}} \nu_{J}(R) &=& \sigma \,(2-q) \, M \frac{R}{H + R} - T \end{array} $$

2 $$\begin{array}{@{}rcl@{}} \nu_{A}(R) &= &\sigma\, q \, M \frac{R}{H + R} - T \end{array} $$

The intake rate of food depends on the mass-specific maximum ingestion rate *M* and the half-saturation density *H*. The parameter *q* modifies the maximum ingestion rate of juveniles and adults; it represents differences in ingestion between life stages in a phenomenological way (de Roos et al. 2008). For *q*<1, juveniles are better at ingesting the resource per unit biomass and have a competitive advantage over adults. For values of *q* higher than 1, the opposite holds. Ingested food is assimilated with conversion efficiency *σ* and first used to cover the mass-specific somatic maintenance costs *T*.

If the assimilated energy is not sufficient to balance the maintenance costs, biomass is lost due to starvation. In addition to starvation, per-capita mortality rates *d*_*J*_(*R*) and *d*_*A*_(*R*) for juveniles and adults, respectively, account for background mortality *μ* and are defined as:

3 $$\begin{array}{@{}rcl@{}} d_{J}(R)&=& \left\{\begin{array}{ll} \mu - \nu_{J}(R), & \text{if} \quad \nu_{J} (R) < 0, \\ \mu, & \text{otherwise}; \end{array}\right. \end{array} $$

4 $$\begin{array}{@{}rcl@{}} d_{A}(R)&=& \left\{\begin{array}{ll} \mu - \nu_{A}(R), & \text{if} \quad \nu_{A} (R) < 0, \\ \mu, & \text{otherwise}. \end{array}\right. \end{array} $$

Under starvation conditions, somatic growth stops and hence no maturation occurs and no energy is invested in reproduction. When the assimilated energy exceeds the maintenance costs, it is invested in somatic growth by juveniles and reproduction by adults. The mass-specific net-biomass production rate of juvenile and adult consumers, restricted to positive values, is indicated with $\nu ^{+}_{J}(R)$ and $\nu ^{+}_{A}(R),$ respectively:

5 $$\begin{array}{@{}rcl@{}} \nu^{+}_{J}(R) &=& \left\{\begin{array}{ll} \nu_{J}(R),& \text{if} \quad \nu_{J} (R) > 0, \\ 0, & \text{otherwise}; \end{array}\right. \end{array} $$

6 $$\begin{array}{@{}rcl@{}} \nu^{+}_{A}(R) &=& \left\{\begin{array}{ll} \nu_{A}(R),& \text{if} \quad \nu_{A} (R) > 0, \\ 0, & \text{otherwise}. \end{array}\right. \end{array} $$

Reproductive energy is stored during the growing season and is transformed all at once to newborn individuals during an annual reproductive event. Seasonal reproduction is thus modeled as a discrete process at regular intervals. Stored reproductive energy is assumed not to increase maintenance costs, following Kooijman in his argument that storage of lipids and fat tissue does not cost any energy (Kooijman 2000). The reproductive energy is stored inside adult individuals and therefore, stored reproductive energy is lost when an adult individual dies.

All consumers are assumed to share the same resource. In the absence of consumers, the resource follows semi-chemostat growth with maximum density *R*_*max*_, and turnover rate *δ*:

7 $$ \frac{dR}{d\tau} = \delta (R_{max} - R). $$

When consumers are present, the resource biomass declines through food ingestion by consumers. Since the resource is explicitly accounted for, density dependence comes about through competition between the individuals via the density of the resource.

### Physiologically structured population model

The physiologically structured population model (PSPM) we use has a cohort structure, like previous PSPMs with seasonal reproduction (Persson et al. 1998). All individuals are born with the same size and growth is deterministic, such that all individuals that are born at the same time remain identical to each other throughout their life. Different from earlier PSPMs with seasonal reproduction, stored energy is not considered reversible; once energy is stored, it cannot be used anymore to cover maintenance costs.

Juveniles are consumers with body size *s* between the size at birth *Sb* and size at maturation *Sm*. Adults do not grow and are thus defined as consumers with body size *s* = *Sm*. The dynamics over time *τ* during the growing season of duration *Y*, (0≤*τ*<*Y*) are all continuous and can be described by the following set of ordinary differential equations:

8 $$\begin{array}{@{}rcl@{}} &\text{for} \quad Sb \leq s_{i} < Sm \text{ :} \\ &\left\{ \begin{array}{l} \displaystyle\frac{d}{d\tau} c_{i}= -d_{J}(R)\,c_{i}, \\ \displaystyle\frac{d}{d\tau} s_{i}= \nu_{J}^{+}(R)\,s_{i}, \\ \displaystyle\frac{d}{d\tau} g_{i} = 0; \end{array} \right. \end{array} $$

9 $$\begin{array}{@{}rcl@{}} &\text{for} \quad s_{i} = Sm \text{ :} \\ &\left\{ \begin{array}{l} \displaystyle\frac{d}{d\tau} c_{i}= -d_{A}(R)\,c_{i}, \\ \displaystyle\frac{d}{d\tau} s_{i}= 0,\\ \displaystyle\frac{d}{d\tau} g_{i}= \nu_{A}^{+}(R)\,Sm. \end{array} \right. \end{array} $$

The number of individuals *c*_*i*_ in each cohort *i* declines with mortality. The positive net-biomass production is invested in somatic growth by juveniles (8) and stored for reproduction in storage *g*_*i*_ by adults (9).

Reproduction of the consumer takes place instantaneously at intervals *t*_*n*_ = *nY*:

10 $$\begin{array}{@{}rcl@{}} &&\left\{\begin{array}{l} c_{0}({t_{n}^{+}}) =\sum \limits_{i =0}^{n}g_{i}(t_{n}^{-})c_{i}(t_{n}^{-})/Sb, \\ s_{0}({t_{n}^{+}}) = Sb, \\ g_{0}({t_{n}^{+}}) = 0;\end{array}\right. \end{array} $$

11 $$\begin{array}{@{}rcl@{}} &&\left\{\begin{array}{l} c_{{i+1}}(t_{n}^{+}) = c_{i} (t_{n}^{-}), \\ s_{{i+1}}(t_{n}^{+}) = s_{i} (t_{n}^{-}), \\ g_{{i+1}}(t_{n}^{+}) = 0. \end{array}\right. \end{array} $$

At the moment just after a reproductive event *t* ^+^, a new cohort is formed from the biomass that was stored up to the moment just before reproduction *t* ^−^ (10). All other cohorts are renumbered, and the biomass of the reproductive storage is set to 0 (11).

Whenever the largest juvenile cohort with index *i* = *m* reaches the maturation size, *s*_*m*_(*t*_*m*_) = *Sm*, at time *t* = *t*_*m*_, a maturation event occurs:

12 $$\begin{array}{@{}rcl@{}} &\left\{\begin{array}{l} c_{i}(t_{m}^{+}) = c_{i} (t_{m}^{-}), \\ s_{i}(t_{m}^{+}) = s_{i} (t_{m}^{-}), \\ g_{i}(t_{m}^{+}) =g_{i}(t_{m}^{-});\end{array}\right. \end{array} $$

13 $$\begin{array}{@{}rcl@{}} &\left\{\begin{array} c_{m} (t_{m}^{+}) = c_{m} (t_{m}^{-}), \\ s_{m}(t_{m}^{+}) = Sm, \\ g_{m}(t_{m}^{+}) =g_{m}(t_{m}^{-}). \end{array}\right. \end{array} $$

At the moment just after a maturation event $t_{m}^{+}$, the largest juvenile cohort becomes an adult cohort, equal in number, size, and storage to the cohort at the moment just before maturation $t_{m}^{-}$ (13). This does not affect any of the other cohorts (12).

Finally, the resource biomass increases through semi-chemostat growth and declines through foraging by consumers:

14 $$\begin{array}{@{}rcl@{}} \frac{d}{d\tau}R &=& \delta (R_{max} - R) - M \, \frac{R}{H+R} \\&&\times \left( (2-q) \, \sum \limits_{i | s_{i} < Sm} c_{i} \, s_{i} + q \, \sum \limits_{i | s_{i} = Sm} c_{i} \, Sm . \right) \end{array} $$

During reproduction and maturation events in the consumer population, the resource density does not change, $R(t_{n}^{+}) = R(t_{n}^{-})$ and $R(t_{m}^{+}) = R(t_{m}^{-})$.

### Stage-structured biomass model

In this section, we derive a stage-structured biomass model from the physiologically structured population model (PSPM) described in the previous section, following an individual-based perspective. We start with the derivation of a consumer population model represented by biomass in the juvenile stage, the adult stage, and the reproductive buffer. In the Appendix, we describe the derivation of a stage-structured biomass model with an adult stage, a reproductive buffer, and multiple juvenile stages instead of just one.

A simplification of the model described in equations (8)–(13) to a model in terms of juvenile biomass, adult biomass, and stored biomass for reproduction implies that we let go of the possibility to track body sizes of individuals through time. Most importantly, this affects the way in which the maturation process is represented in the stage-structured biomass model. In the PSPM, the maturation of each cohort occurs as a discrete event whenever it reaches the size at maturation. In the stage-structured model, we assume instead that individuals mature from the juvenile to the adult stage (or to the next juvenile stage in case of multiple juvenile stages; see Appendix) at a per-capita rate that is independent of their body size. To nonetheless preserve the connection with individual-level processes, we derive an expression for the maturation rate that ensures that, for a given resource density, the expected lifetime contribution of biomass per individual to the adult phase is the same as in the PSPM. We show the consequences of this approximation for the transition of biomass from the juvenile to the adult stage in the Appendix.

With a constant per-capita maturation rate for juveniles, the continuous dynamics over time *τ* during the growing season of duration *Y*, (0≤*τ*<*Y*) for juvenile cohorts becomes:

15 $$ \left\{ \begin{array}{l} \displaystyle\frac{d}{d\tau} c_{i}= -d_{J}(R)\,c_{i} - \gamma \, c_{i}, \\ \displaystyle\frac{d}{d\tau} s_{i}= \nu_{J}^{+}(R)\,s_{i}, \\ \displaystyle\frac{d}{d\tau} g_{i} = 0; \end{array} \right. $$

The crucial difference between the system of equations above and Eq. 8 is the size-independent, per-capita maturation rate *γ*, which is introduced to derive the stage-structured biomass model. The biomass in the juvenile stage *J* of the stage-structured biomass model can be defined as:

16 $$ J (\tau)= \sum \limits_{i} c_{i} \, s_{i}, $$

where the summation is over all juvenile cohorts. Using Eq. 15, this definition of *J* (16) leads to the following ODE for the change in total juvenile biomass over time:

17 $$\begin{array}{@{}rcl@{}} \frac{d}{d\tau}J \, &=& \, \nu_{J}^{+}(R) \sum\limits_{i} c_{i} \, s_{i} -d_{J}(R) \sum \limits_{i} c_{i} \, s_{i} - \gamma \sum \limits_{i} c_{i} \, s_{i} \\ &=& (\nu_{J}^{+}(R) -d_{J}(R) - \gamma) J. \end{array} $$

For the derivation of an expression for the maturation function *γ* that preserves the expected lifetime contribution of biomass by an individual to the adult stage from the PSPM, we assume a constant resource density that is high enough for the juvenile ingestion rate to exceed the maintenance requirements. Under these circumstances, the net-biomass production rate *ν*_*J*_(*R*) is constant and positive. To simplify the notation, we express this rate as *ν*_*J*_. In addition, no mortality due to starvation occurs and *d*_*J*_(*R*) hence equals *μ*_*J*_.

First, we need to determine the probability of maturation of an individual in the PSPM. Following Eq. 8, the body mass of an individual increases with age *a* from the size at birth as $s (a ) ={ Sb}\,{\mathrm {e}^{{ \nu _{J}} a}}$. The duration of the juvenile stage depends on the time it takes to grow from the size at birth to the size at maturation. Based on the change in body size with age, the age at maturation equals:

18 $$ a_{m}=\frac{\ln ({\frac {{ Sm}}{{Sb}}} )}{ { \nu_{J}}}= \,{\frac {-\ln ({z} ) }{{ \nu_{J}}}}, $$

where we introduce the parameter *z* to reflect the ratio between size at birth and maturation, *z* = *Sb*/*Sm*. The survival probability *p*(*a*) of a juvenile individual depends only on mortality (8), and, the survival probability as a function of age thus follows $p (a ) ={\mathrm {e}^{-\mu _{J}\,a}}$. Using these definitions of *a*_*m*_ and *p*(*a*), the probability of maturation of an individual becomes:

19 $$ p(a_{m}) \, = \,{z}^{{\frac {\mu_{J}}{{ \nu_{J}}}}}, $$

and the expected amount of biomass each individual contributes to the adult stage thus equals:

20 $$ Sm\,p(a_{m}) \, = Sm\,{z}^{{\frac {\mu_{J}}{{ \nu_{J}}}}}. $$

In the stage-structured biomass model, the juvenile biomass as a function of time from initial density *J*_0_ follows from ODE (17):

$$J(t)\, = \, {J_{0}}\, \mathrm{e}\,^{(\nu_{J} -\mu_{J} -\gamma )\, t}, $$

and the biomass that matures as a function of time is:

$$\gamma \,J(t)\, = \, \gamma {J_{0}}\, \mathrm{e}\,^{(\nu_{J} -\mu_{J} -\gamma )\, t}. $$

The expected lifetime contribution of biomass of one individual (*J*_0_ = *Sb*) to the adult stage in the stage-structured biomass model thus equals:

$${\int}_{0}^{\infty }\! {\gamma}\, Sb \, \mathrm{e}\,^{(\nu_{J} -\mu_{J} -\gamma )\, t} \; \mathrm{d}t\, = \, \lim_{t \to \infty} \frac{\gamma \, Sb\, (\mathrm{e}\,^{(\nu_{J} -\mu_{J} -\gamma )\, t} - 1)}{(\nu_{J} - \mu_{J} - \gamma)}. $$

As the sum of maturation and mortality should always exceed the growth over the juvenile period of an individual the limit at the right-hand side can be simplified to −*Sb* *γ*/(*ν*_*J*_−*μ*_*J*_−*γ*).

After equating the latter expression with Eq. 20, we find the following expression for *γ*:

21 $$ \gamma (\nu_{J}, \mu_{J}) = \dfrac { \nu_{J} - \mu_{J}} {1-z^{1-\frac{\mu_{J}}{\nu_{J}}}}. $$

This is the same maturation function as for the stage-structured biomass model with continuous reproduction (de Roos et al. 2008). The function approaches 0 for *ν*_*J*_ *↓*0, and never reaches negative values, while it has a regular limit equal to −*μ*_*J*_/*ln*(*z*) for $\nu _{J} \rightarrow \mu _{J}$. So far, we considered a situation with abundant resources in which the net-biomass production is positive. Now, we reintroduce the food-dependence of the net-biomass production and mortality rates. In case the resource density is constant but low, and food-ingestion does not cover the maintenance costs, growth stops, and no maturation takes place:

22 $$ \gamma (\nu_{J}(R), d_{J}(R)) = \left\{\begin{array}{ll} \displaystyle\frac { \nu_{J} (R) - d_{J}(R)} {1-z^{1-\frac{d_{J}(R)}{\nu_{J}(R)}}}, & \text{if} \quad \nu_{J} (R) > 0, \\ 0, & \text{otherwise}. \end{array}\right. $$

While maturation of biomass follows a delta function over time in the PSPM (all individuals mature at once), the introduction of a size-independent per-capita maturation function implies that it follows an exponential distribution over time in the stage-structured biomass model. We compare the contribution of biomass to the adult stage over time for the PSPM and stage-structured biomass model in the Appendix. We also show there that in a stage-structured biomass model with multiple juvenile stages, maturation of biomass follows a gamma distribution over time (Appendix).

The choice for a per-capita size-independent maturation rate of juveniles has the consequence that adults are no longer characterized by the same body size. Nonetheless, total biomass in the adult stage *A* equals the product of the density and body size of all adult individuals. An ODE describing the dynamics of *A* can be derived from equation (9) and the ODE (17) derived for *J*. Maturation of juvenile biomass results in an increase of the adult biomass with the same rate. Once individuals reach the adult stage, they do not grow any further. All individuals in the adult stage suffer from mortality as the adults in the PSPM do (9). Since this per-capita rate is assumed equal for all individuals, it can be directly used as a mass-specific mortality rate. This results in the following dynamics for *A*:

23 $$ \frac{d}{d\tau}A= \gamma(\nu_{J}(R), d_{J}(R)) J - d_{A}(R)A. $$

The total biomass *B* stored by all adult individuals for reproduction equals the product of the adult density and their individual reproductive storage *g*_*i*_. From equation (9) for *g*_*i*_, it can be derived that *B* increases with the net-biomass production of adults. In addition, *B* decreases with the same mortality rate as the adults because when an adult individual dies its reproductive storage perishes as well. The ODE for *B* thus equals:

24 $$ \frac{d}{d\tau}B=\nu_{A}^{+}(R)A - d_{A}(R)B. $$

In summary, the derivation above results in the following system of ordinary differential equations as a function of time for the dynamics of juvenile, adult, and reproductive storage biomass in the interval between reproductive events, *Y*, (0≤*τ*<*Y*):

25 $$\begin{array}{@{}rcl@{}} \frac{dJ}{dt}& =& \nu^{+}_{J}(R) J - \gamma (\nu_{J}(R), d_{J}(R)) J - d_{J}(R) J \end{array} $$

26 $$\begin{array}{@{}rcl@{}} \frac{dA}{dt} & = &\gamma \, (\nu_{J}(R), d_{J}(R)) J - d_{A}(R) A \end{array} $$

27 $$\begin{array}{@{}rcl@{}} \frac{dB}{dt} &=& {\nu^{+}_{A} (R)} {A} - d_{A}(R) {B} \end{array} $$

28 $$\begin{array}{@{}rcl@{}} \frac{dR}{dt} & = &\delta \, (R_{max} - R) - ((2-q) J + q\,A) \,M \frac{R}{H + R}\,. \end{array} $$

As in the PSPM (10, 11), seasonal reproduction takes place instantaneously at intervals *t*_*n*_ = *nY*:

29 $$\begin{array}{@{}rcl@{}} J(t_{n}^{+})&=&J(t_{n}^{-}) + B(t_{n}^{-}) \end{array} $$

30 $$\begin{array}{@{}rcl@{}} A(t_{n}^{+})&=&A(t_{n}^{-}) \end{array} $$

31 $$\begin{array}{@{}rcl@{}} B(t_{n}^{+})&=&0 \end{array} $$

32 $$\begin{array}{@{}rcl@{}} R(t_{n}^{+})&=&R(t_{n}^{-})\,. \end{array} $$

The process is exactly the same as for the PSPM, without the renumbering of the cohorts. At a reproductive event, the densities of adult consumer and resource biomass do not change (30, 32). The juvenile biomass increases with the biomass in the reproductive storage (29), and the biomass of the reproductive storage is set to 0 (31).

In the results section, we compare the model dynamics of the stage-structured biomass model we derived (25)–(32) with the PSPM described in equations (8)–(14). Moreover, we investigate the consequences of a gamma distribution instead of an exponential distribution for maturation of biomass over time by comparing the dynamics of a stage-structured biomass model with three juvenile stages (39)–(50), instead of one, with the PSPM (8)–(14).

### Parameterization and calculations

Parameterization follows the approach laid out by de Roos and Persson (2013, Box 3.4) Table 1 lists default values for all parameters. The mass-specific rates for metabolism *T* and maximum ingestion *M* and the per-capita background mortality *μ* are derived from scaling relationships with a characteristic adult body size. The half-saturation density *H* is defined as the ratio of an individual’s maximum ingestion and attack rate. Across differently sized species, these rates are both assumed to scale with body mass to a power of 3/4 (de Roos and Persson 2013). This makes the half-saturation density independent of individual mass as well as species independent. The value of *q* sets the difference in the maximum ingestion rate between juvenile and adult consumers. It can not be derived from experimental data as it is a phenomenological representation of a combination of factors. We tested populations where the juveniles, (*q* = 0.5) or the adults (*q* = 1.5) are competitively superior or performance of the two is the same (*q* = 1) (see de Roos et al. 2013, for an extensive study on the effect of *q* in case of continuous reproduction). The assimilation efficiency *σ* is an estimate independent of body size. The size at maturation *Sm* always equals the average adult body size *W*_*A*_ because adults are assumed not to increase in somatic mass. The relationship of the body size at birth *Sb* with average adult body size varies across different species (de Roos and Persson 2013). We used a ratio between the size at birth and size at maturation $z = \frac {Sb}{Sm}$ of 0.1, which lies in the range of values normally found across species.

**Table 1:** Default parameter values (de Roos and Persson 2013, Box 3.4)

Symbol	Unit	Description	Value
*M*	day ^−1^	Mass-specific maximum ingestion rate	0.1$\,{W_{A}}^{{-0.25}}$
*T*	day ^−1^	Mass-specific maintenance rate	0.01$\,{W_{A}}^{{-0.25}}$
*μ*	day ^−1^	Background mortality rate	0.0015$\,{W_{A}}^{{-0.25}}$
*W*_*A*_	g	Characteristic adult body size	50
*H*	mg L ^−1^	Half-saturation resource density	3
*q*	–	Competitive difference between stages	1, 1.5 or 0.5
*σ*	–	Assimilation efficiency	0.5
*z*	–	Ratio size at birth to size at maturation	0.1
*Y*	days	Year, period between reproductive events	250
*Sm*	g	Body size at maturation	*W*_*A*_
*Sb*	g	Body size at birth	*z* *Sm*
*R*_*max*_	mg L ^−1^	Resource maximum density	20
*δ*	day ^−1^	Resource turnover rate	0.1

We based our parameterization for the consumer on invertebrate species with a range of average adult body sizes *W*_*A*_ between 0.02 and 200 g. In the absence of starvation mortality, the average lifetime in our consumer population equals 1/*μ*, and, the average adult body size thus determines the average lifetime of the consumer under these circumstances. This results in an average lifetime of between 250 and 2500 days for our parameter settings (Table 1). The length of the year is set to *Y* = 250 days because we assume that during winter all processes slow down to negligible rates. Varying *W*_*A*_ between 0.02 and 200 g, consumers thus experience between 1 and 10 reproductive events over a lifetime on average. In addition, the average adult body size scales all energetic processes such that species characteristics vary over a classic fast-slow spectrum. Species that are small have short lifetimes, few reproductive events, and high mass-specific energetic rates; species that are large live long and have many reproductive events but low mass-specific energetic rates.

We are mostly interested in the dynamics of the consumer, and parameters for the resource are therefore chosen such that consumer persistence is ensured. The maximum resource density is set at a value which is about an order of magnitude larger than the half-saturation density of the consumer. For the resource turn-over rate, we chose a value of 0.1.

Population dynamics are studied using numerical simulations. The parameter dependence of the dynamics was studied with integrations for a given parameter combination over long time periods, of 200,000 days, while varying the value of a parameter in small steps between consecutive periods of 200,000 days (see de Roos and Persson 2013, box 3.5). A period of 200,000 days or 800 years results in simulations over 80 generations for the largest species we considered (given their average lifetime of 2500 days). This should be sufficiently long for the dynamics to stabilize. Average output values were calculated in such bifurcation runs over the last 60 *%* of the 200,000-day period. The biomasses just after reproduction are plotted for the last 40 years, or last 10,000 days of these 200,000 days. We investigated the effect of species body size and the number of juvenile stages in the stage-structured biomass model on the similarity of the dynamics of the stage-structured biomass model and the PSPM.

## Results

### The contribution of biomass to the adult stage over time

**Fig. 1:**
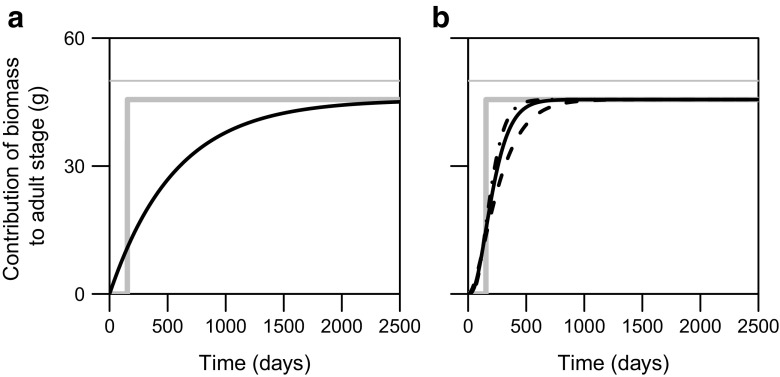
Comparison of the cumulative contribution of biomass to the adult stage over time by a newborn individual in the physiologically structured population model (*gray-thick line*) and the stage-structured biomass model with **a** one juvenile stage (in *black*), and **b** two (*black-dashed line*), three (*black-solid line*), and four (*black-dash-dotted line*) juvenile stages. Also, the body mass of an adult individual (*W*_*A*_; *gray-thin line*) is indicated in the graphs. Default parameter values and *ν*_*J*_ = 0.015, which is the maximum value for the net-biomass production of juveniles when *q* = 1, were used. The functions for the cumulative amount of biomass in the adult stage over time correspond to Eqs. 33, 35 and 37, calculated in the Appendix

The expected lifetime contribution of biomass by a single newborn individual to the adult stage of the physiologically-structured population model (PSPM) is, for a given resource density, preserved in the stage-structured biomass model (Fig. 1). The main difference between the two models relates to the cumulative contribution of biomass over time to the adult stage by a single newborn individual (Appendix, Fig. 1a). While maturation of biomass follows a delta function over time in the PSPM (all individuals mature at once), the introduction of a size-independent per-capita maturation function leads to an exponential distribution over time in the stage-structured biomass model. This can be interpreted as such that some individuals may mature earlier or later, at smaller or larger body sizes, than they would do in the PSPM. The maturation function derived here does preserve that maturation is a process that depends on the individual energy assimilation and juvenile mortality (see Eq. 22).

In a stage-structured biomass model with multiple juvenile stages, maturation of biomass follows a gamma distribution over time (Appendix, Fig. 1b). This results in a lower maturation probability just after birth than for a stage-structured biomass model with one juvenile stage and a maturation probability over time that becomes more similar to the PSPM (compare Fig. 1a with Fig. 1b). The maturation of biomass in a stage-structured biomass model with three juvenile stages closely resembles the probability distribution in case of more than three juvenile stages (Fig. 1). We therefore compare, in Section 1, numerical simulations for a stage-structured biomass model with one as well as three juvenile stages with the PSPM.

### Impact of stage-dependent energetics

**Fig. 2:**
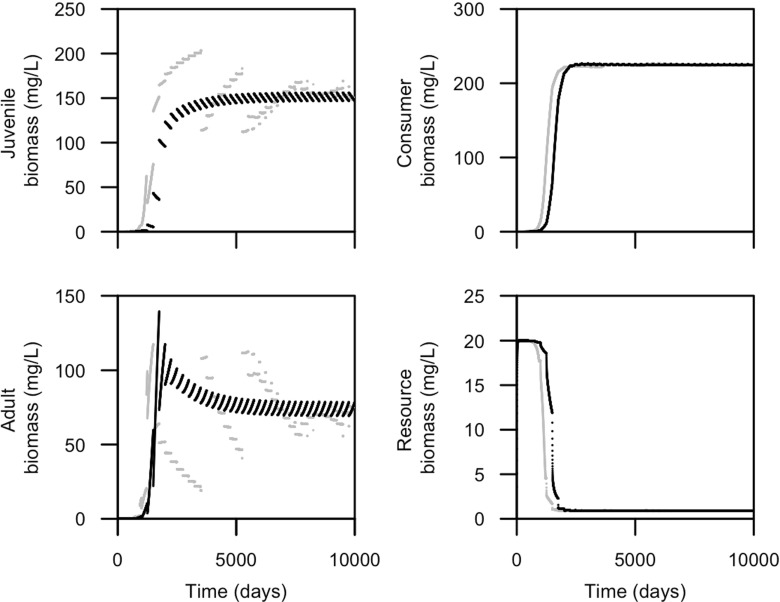
Dynamics over time of consumer populations with juveniles and adults that are equal competitors for the resource (*q* = 1, see Table 1 for other parameter values). Juvenile (*top left*), adult (including storage; *bottom left*), total consumer (*top right*), and resource biomass (*bottom right*) for the physiologically structured population model (in *grey*) and stage-structured biomass model (in *black*) are shown.

Figure 2 compares the dynamics over time of the PSPM with a stage-structured biomass model with one juvenile stage and for the case that juveniles and adults are energetically equal. Reproductive energy is stored over the season and released at the beginning of each year, so each 250 days. The dynamics of both models show instantaneous changes in juvenile and adult biomass due to reproduction. In addition, the PSPM shows instantaneous changes in juvenile and adult biomass due to maturation events. The more gradual changes in biomass over time in both models stem from mortality and increasing stored biomass for adults and somatic growth and mortality for juveniles. Eventually, the dynamics of both models converge to regular, seasonal oscillations, and the biomass of the juveniles and adults and the total consumer population and resource in the two models become very similar (Fig. 2).

**Fig. 3:**
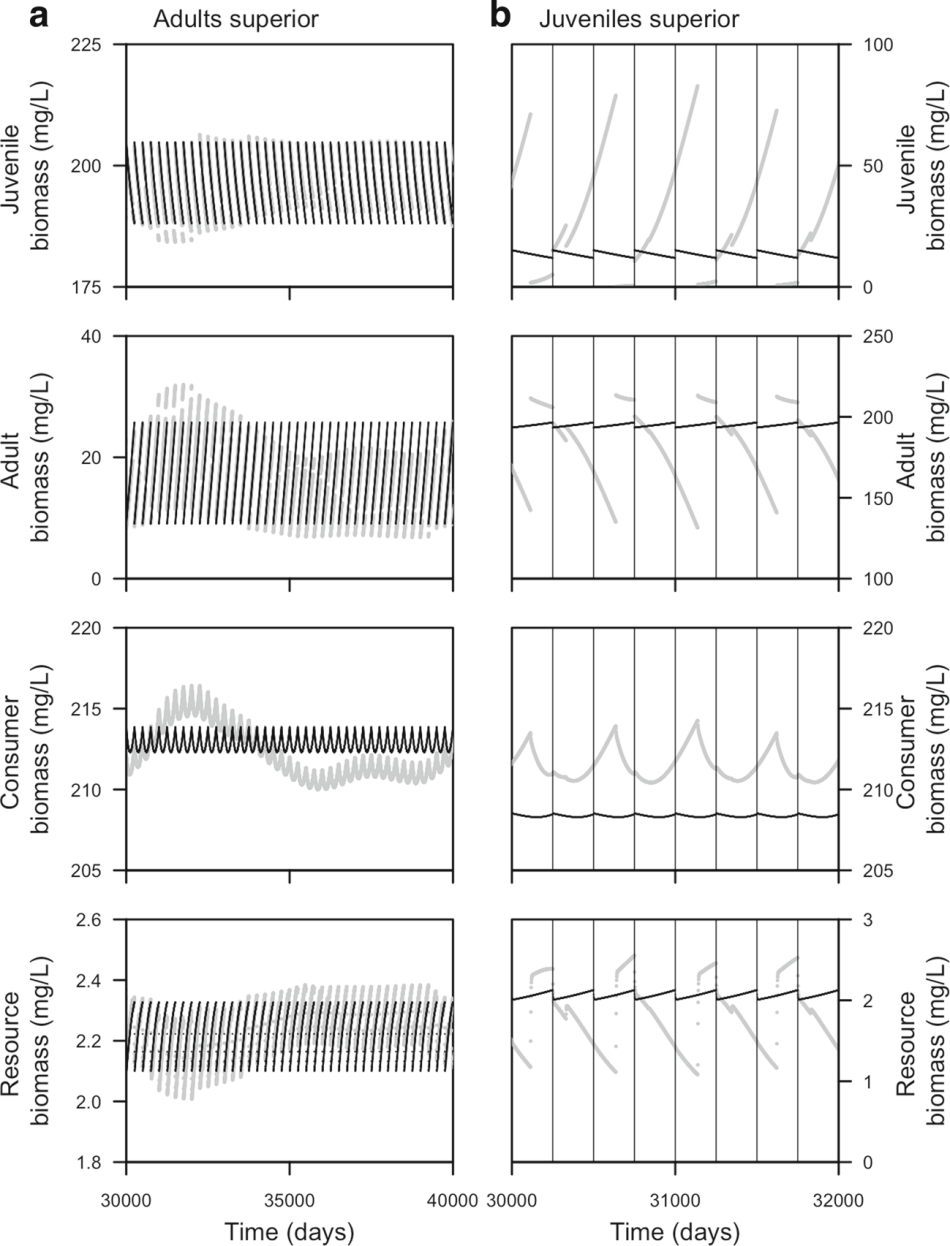
Dynamics over time of consumer populations with either **a** adults (*q* = 1.5) or **b** juveniles (*q* = 0.5, see Table 1 for other parameter values) having a higher resource ingestion rate per unit biomass. Juvenile, adult (including storage), total consumer, and resource biomass for the physiologically structured population model (in *gray*) and stage-structured biomass model (in *black*) are shown. In addition, times at which reproductive events occur are plotted in the right panels (using vertical *black-thin lines*)

Population cycles occur in the PSPM when juveniles and adults are energetically different from each other (Fig. 3). These cycles, so-called adult-driven cycles when adults have a higher, mass-specific food-ingestion rate and so-called juvenile-driven cycles when juveniles have a higher, mass-specific food-ingestion rate, were earlier described by de Roos and Persson (2003). Adult-driven cycles are characterized by slow oscillations, with low adult biomass and high juvenile biomass. Since the adults are more energy efficient, the reproductive output is high and reproduction occurs each year while juvenile growth and maturation is slow. On the other hand, juvenile-driven cycles are characterized by large amplitude fluctuations in juvenile and adult biomass in which a single cohort of consumers dominates the population dynamics throughout its life. While in the juvenile phase, the dominating cohort may suppress reproduction, leading to years with low (Fig. 3) or even without (see Fig. 7b) reproduction. Even though the stage-structured biomass displays fixed point dynamics and no population cycles, the biomass densities over time do not deviate much between the two models when the PSPM displays adult -driven cycles (Fig. 3). When the PSPM displays juvenile-driven cycles, the two models deviate because the stage-structured biomass model does not show large amplitude cycles in juvenile and adult biomass.

**Fig. 4:**
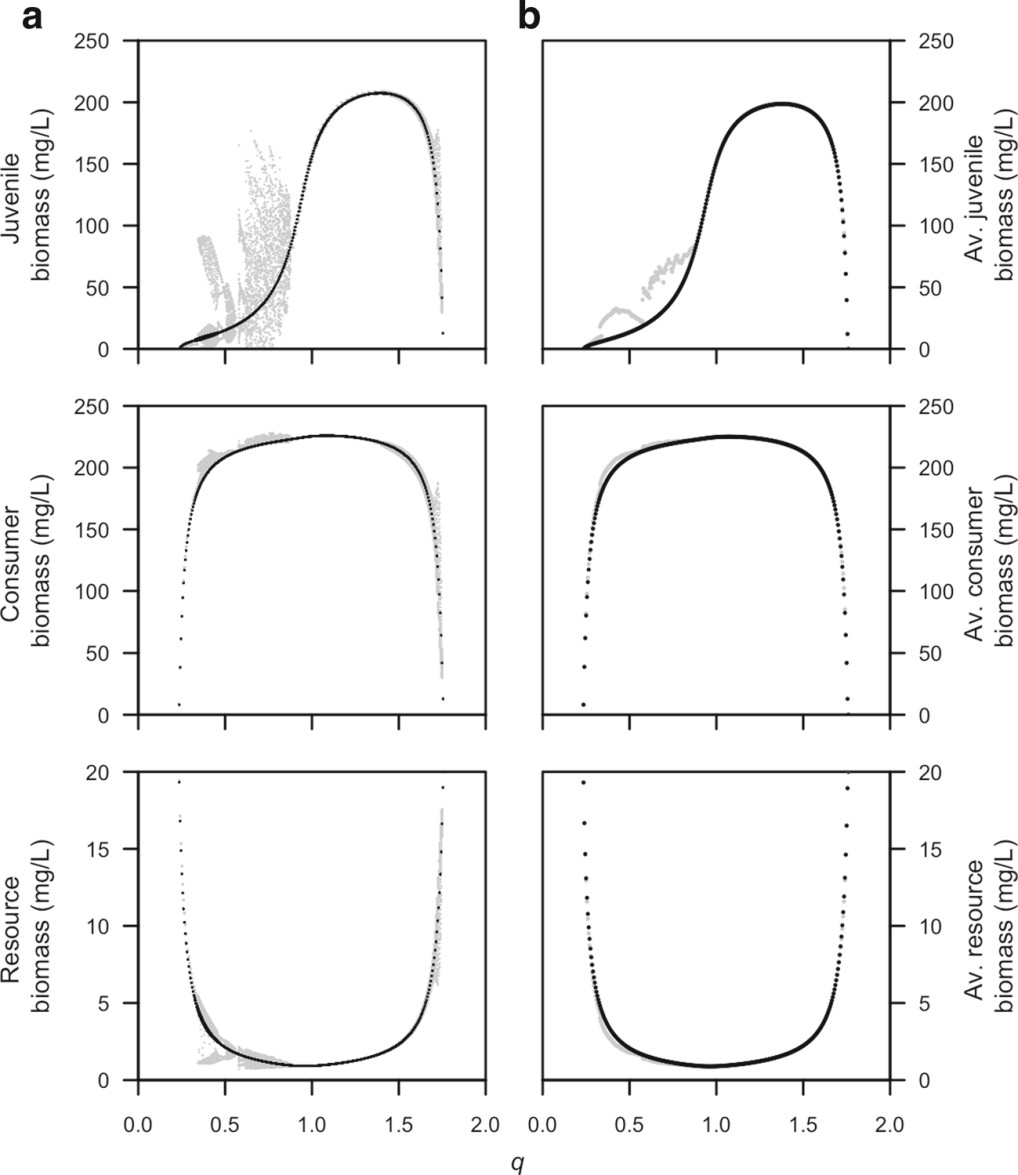
Juvenile, total consumer, and resource biomass are plotted in relation to different values of the competitive difference between juveniles and adults *q* (see Table 1 for other parameter values). Both **a** biomass just after reproduction and **b** time-average biomass densities are plotted for the physiologically structured population model (in *gray*) and stage-structured biomass model (in *black*)

Figure 4 illustrates the extent of the occurrence of population cycles for different values of *q*, the parameter that scales the differences in mass-specific food-ingestion rates between adults and juveniles. While the time dynamics between the two models deviate from each other, the average biomass densities over time of the two models are almost identical for high values of *q* (*q* = 0.9−1.8; adults have a higher, mass-specific food-ingestion rate). On the other hand, for low values of *q* (*q* = 0.4−0.9; juveniles have a higher, mass-specific food-ingestion rate), the average biomasses deviate between the models (Fig. 4). In case of continuous reproduction of consumers, a comparison of the PSPM with its corresponding stage-structured biomass model shows the same deviations in the dynamics when juveniles are superior foragers (de Roos et al. 2008).

### Species body size

**Fig. 5:**
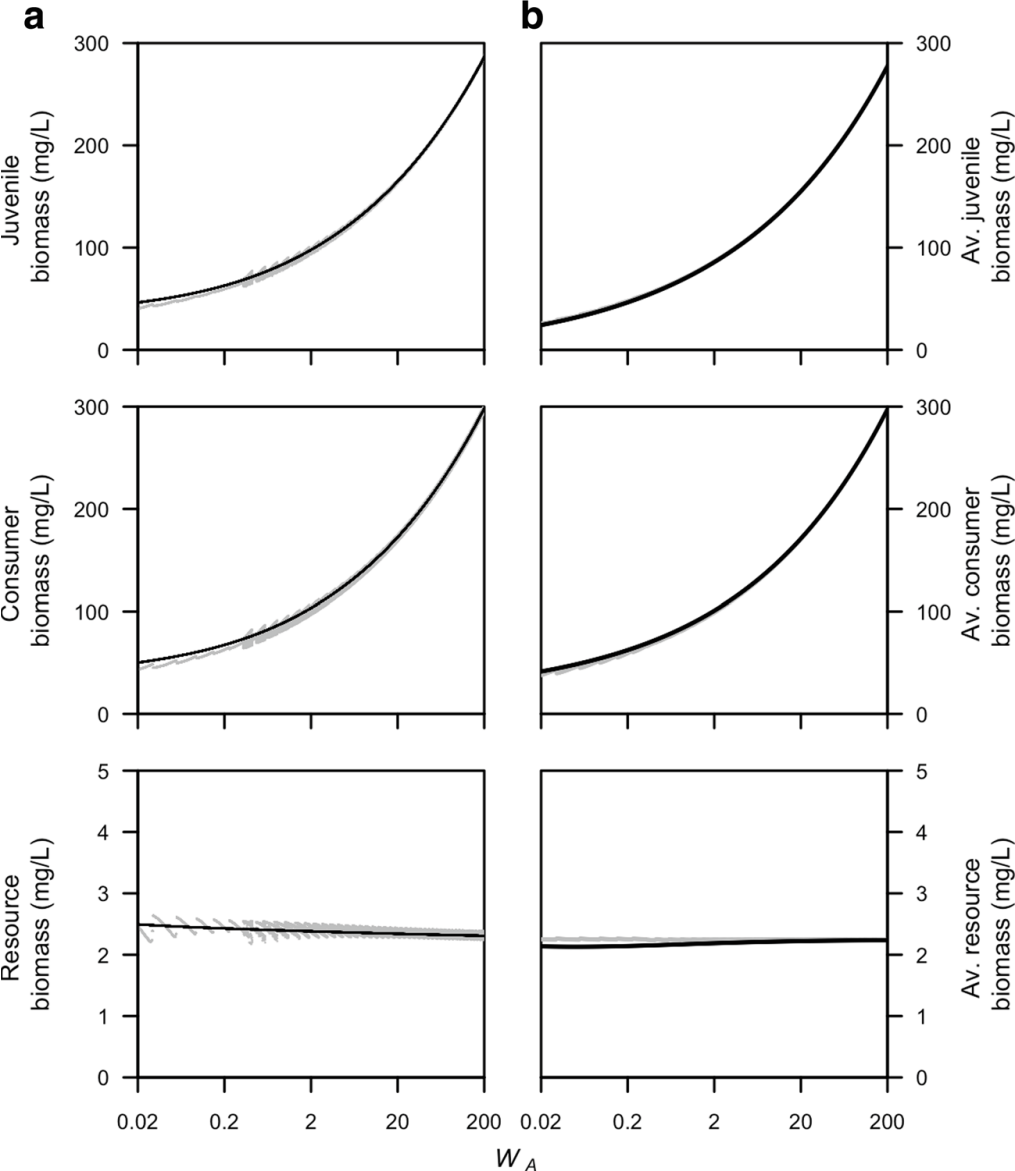
Juvenile, total consumer, and resource biomass are plotted in relation to different values of the average adult body mass *W*_*A*_ for consumer populations with adults that are superior at ingesting the resource (*q* = 1.5, see Table 1 for other parameter values). Both **a** biomass just after reproduction and **b** time-average biomass densities are plotted for the physiologically-structured population model (in *gray*) and stage-structured biomass model (in *black*)

**Fig. 6:**
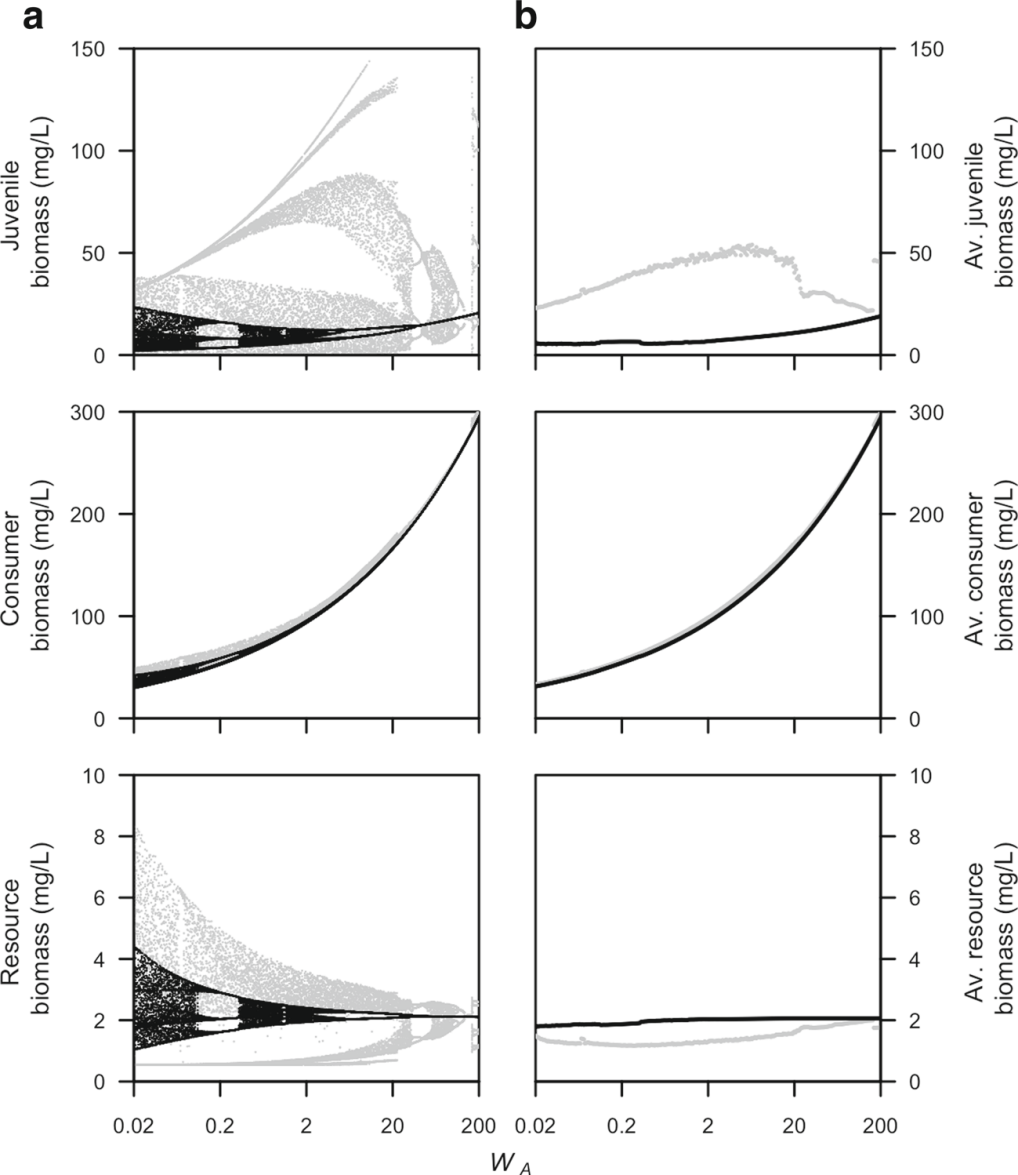
Juvenile, total consumer, and resource biomass are plotted in relation to different values of the average adult body mass *W*_*A*_ for consumer populations with juveniles that are superior at ingesting the resource (*q* = 0.5, see Table 1 for other parameter values). Both **a** biomass just after reproduction and **b** time-average biomass densities are shown for the physiologically structured population model (in *gray*) and stage-structured biomass model (in *black*)

Figures 5 and 6 show that with a larger species body size, the total biomass increases. This is due to the longer lifetime that results from the lower mortality rate at a larger body size and the decrease in mass-specific maintenance rate with body size. In addition, the change in energetic rates with increasing body size result in a change in the duration of the juvenile stage. This also results in discrete transitions in the increasing biomass whenever the maturation event shifts from just before to just after the reproductive event in the PSPM.

When adults are competitively superior (Fig. 5), the juvenile and resource biomass just after reproduction of the two models are nearly identical over the whole range of species body sizes. The adult biomass just after reproduction in the PSPM oscillates around that of the stage-structured biomass model. In contrast, the time-average biomass densities are always very similar in the two models. For small body sizes, the population cycles in the PSPM disappear, but this does not improve the match between the two models. When juveniles are superior at ingesting the resource (Fig. 6), cycles occur for the whole range of body sizes in the PSPM. The stage-structured biomass model also shows cycles for small body sizes, but the oscillations generally have a smaller amplitude than in the PSPM. Due to the cycles, the average biomass densities deviate between the two models, especially at intermediate species body sizes (*W*_*A*_ = 0.2−20 gram).

### Stage-structured biomass model with multiple juvenile stages

**Fig. 7:**
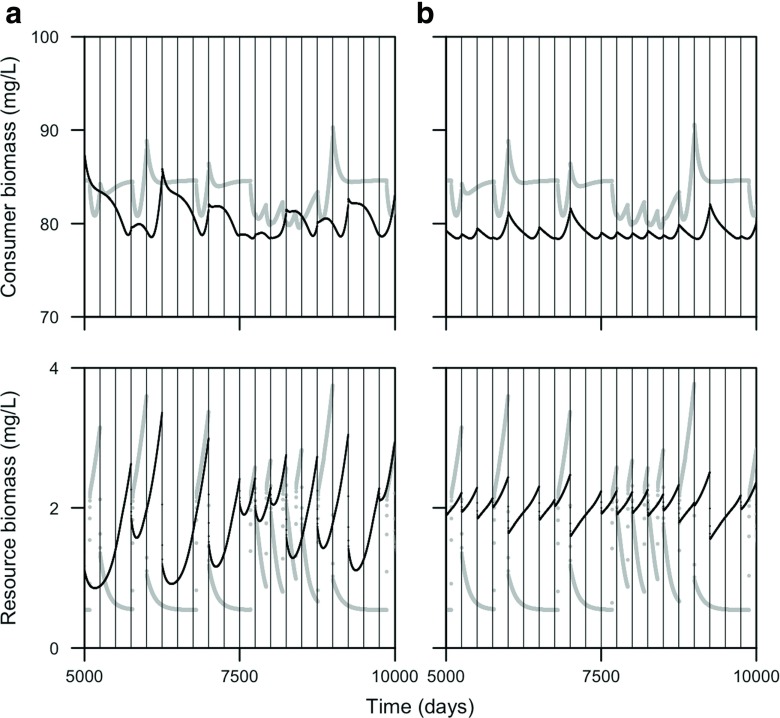
Consumer and resource biomass is plotted over time for the physiologically-structured population model (in *gray*) and stage-structured biomass model (in *black*) with either **a** three juvenile stages or **b** one juvenile stage. In addition, times at which reproductive events occur are shown (using vertical *black-thin lines*). The body mass of an adult consumer is set equal to *W*_*A*_ = 1 g and juvenile consumers are superior at ingesting the resource (*q* = 0.5, see Table 1 for other parameter values)

Figure 7 shows that, when juveniles are superior at ingesting the resource, the stage-structured model with three juveniles stages shows cycles with a longer period, more similar to the PSPM, than the model with one juvenile stage (model equations for the stage-structured biomass model with three juvenile stages can be found in the Appendix). Periods without reproduction may last up to 2 years in the stage-structured model with three juvenile stages (note that a reproductive event results in a sudden drop of the resource biomass density, Fig. 7). In the stage-structured model with one juvenile stage on the other hand, a period without reproduction may maximally last for 1 year. This difference is probably due to the different maturation rate, the contribution of biomass to the adult stage of a juvenile cohort is more concentrated in time with three juvenile stages than with one (see Fig. 1). Still, the stage-structured biomass model never displays a discrete separation in time between adults and juveniles such as can be observed in the PSPM (not shown).

**Fig. 8:**
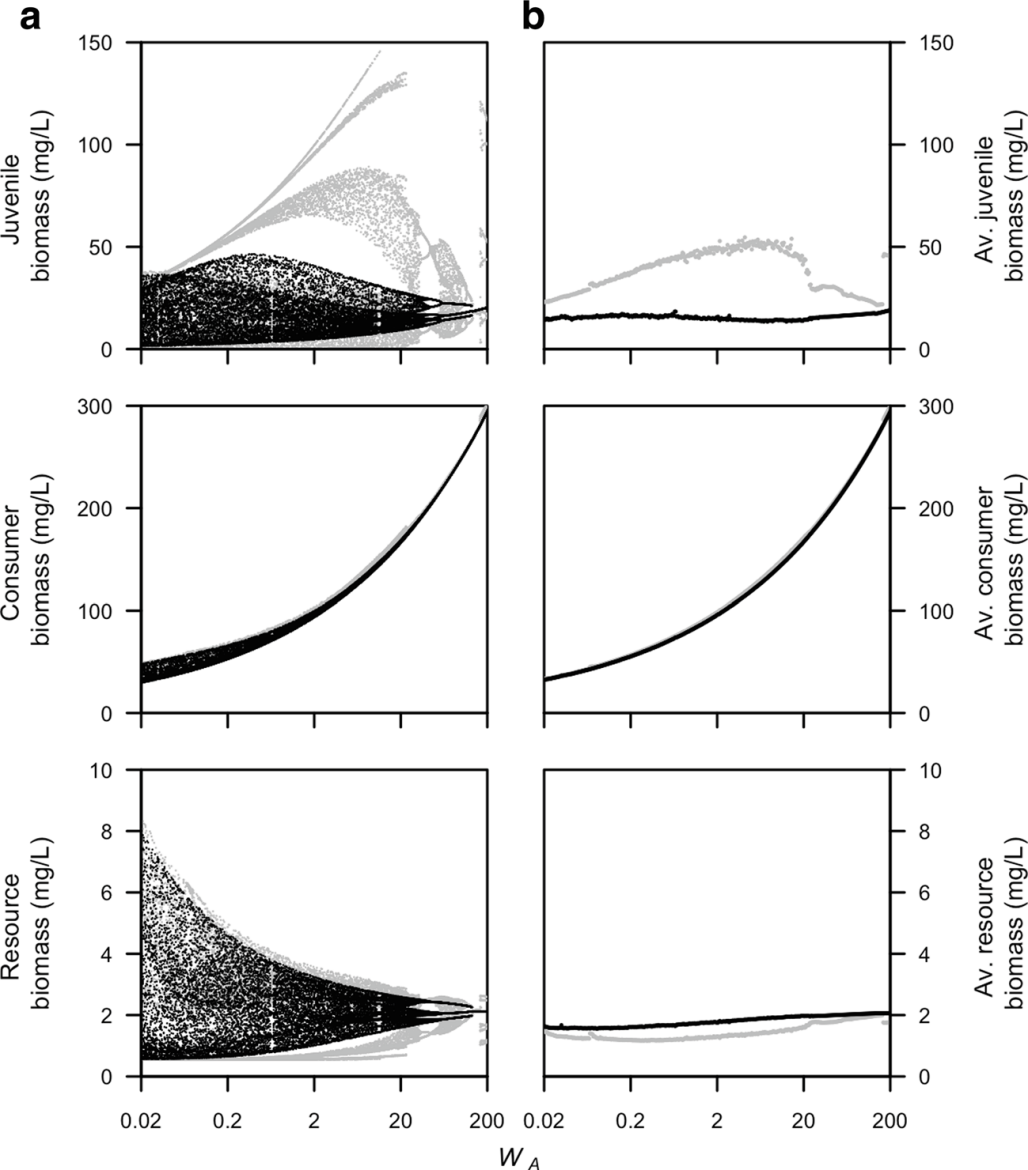
Juvenile, total consumer, and resource biomass are plotted in relation to different values of the average adult body mass *W*_*A*_ for a stage-structured biomass model with three juvenile stages. Both **a** biomass just after reproduction and **b** time-average biomass densities for the physiologically structured population model (in *gray*) and stage-structured biomass model with three juvenile stages (in *black*). The figure is directly comparable to Fig. 6, which shows a stage-structured biomass model with one juvenile stage for the same parameter values. Juvenile consumers are superior at ingesting the resource (*q* = 0.5, see Table 1 for other parameter values). Model equations for the stage-structured biomass model with three juvenile stages can be found in the Appendix

Population cycles occur over a wider range of body sizes in a stage-structured biomass model with three juvenile stages than for the model with one juvenile stage (compare Fig. 8a with Fig. 6a). In addition, the model with three juvenile stages always displays oscillations with a larger amplitude than the model with one juvenile stage does. In addition, the average biomass densities of the model with three juvenile stages lies closer to the average biomass densities of the PSPM (compare Fig. 8b with Fig. 6b). Yet, the average biomass densities of adults and juveniles of the two models still deviate over a range of parameter values (Fig. 8b). Multiple juvenile stages in the stage-structured biomass model do not affect the close match with the PSPM when adults are superior at ingesting the resource that already occurred with a single juvenile stage (not shown).

### Computational implications of the model simplifications

The number of cohorts that is present in the population at one time in the PSPM may increase up to around 140 over time (not shown). Since per cohort, three ODEs are used to track the dynamics (one for the number of individuals, one for the body size, and one for the reproductive storage), calculations in the PSPM may require up to 420 ODEs simultaneously. For the consumer in the stage-structured biomass model, three ODEs are used in the case of a single juvenile stage and five ODEs are used in the case of three juvenile stages. As a result, the bifurcation run shown in Fig. 4 takes about 40 h for the PSPM, while the same bifurcation with the stage-structured biomass model is completed in about 3 h.

## Discussion

The stage-structured biomass model with seasonal reproduction presented in this manuscript is derived from a cohort-based, physiologically structured population model (PSPM) with seasonal reproduction. The model is a consistent translation of individual-level assumptions about food ingestion, bioenergetics, growth, investment in reproduction and storage of reproductive energy to stage-based processes at the population level. The maturation function in the stage-structured biomass model spreads out the discrete maturation of a cohort in the PSPM over time, but preserves the expected lifetime contribution of biomass of an individual to the adult phase. Numerical simulations of the two models show similar values for the average biomasses over time. In the case of intermediate body sizes and a competitive advantage for juveniles large amplitude population cycles occurring in the PSPM cause the models to deviate. The inclusion of multiple juvenile stages leads to population dynamics in the stage-structured biomass model that are more similar to the PSPM. The stage-structured biomass modeling framework that we derived allows for analysis of size-structured populations with seasonal reproduction using a relatively simple set of ODEs.

The stage-structured model presented here assumes that the consumer’s reproduction is bound to a specific season, while the resource reproduces continuously. In addition, foraging, background mortality, growth, investment in reproduction, and maturation in the consumer are assumed to occur continuously between reproductive events. These factors result in a dynamical complexity that cannot easily be caught in discrete-time equations but require “semi-discrete” consumer dynamics (Pachepsky et al. 2008; Murdoch et al. 2003). In the model, reproduction is assumed to be an externally forced, seasonal event that occurs simultaneously for all mature individuals. Timing of spawning has been associated with seasonal external cues such as day length, food quality, humidity, solar insolation, and temperature (Bromage et al. 2001; Rubenstein and Wikelski 2003; Brown and Shine 2006; van Woesik et al. 2006). The seasonal species in our study are in essence capital breeders that save reproductive energy during the growing season prior to reproduction. This is considered a common life-history strategy in seasonal species (Bonnet et al. 1998; McBride et al. 2013).

So far, models that consider population size-structure, seasonal reproduction, an explicit growing season, and individual-level energetics are relatively complex physiologically structured population models (Persson et al. 1998; Claessen et al. 2000; van de Wolfshaar et al. 2008; van Leeuwen et al. 2013). PSPMs can theoretically result in calculations with an unlimited number of ODEs to describe the cohorts that are at one time present in the population. The stage-structured biomass model presented here consists of a limited set of ODEs (five ODEs for the model with three juvenile stages). Numerical bifurcations such as shown in the results section of this manuscript require only one tenth of the computational time of the PSPM using the stage-structured biomass model. The complexity of the PSPMs has hindered the study of large species ensembles with a size-dependent energetics approach. This is illustrated by the fact that so far published studies with PSPMs describe maximally two interacting size-structured populations. While the model presented here is a consumer-resource model, it is relatively straightforward to extend the model to multiple interacting species. A similar modeling framework to the one presented here, but for cases with continuous reproduction, has been used to study the sole-plaice interaction (van de Wolfshaar et al. 2012) and cod-herring interaction (van Denderen and van Kooten 2013) in the North Sea and the sprat-cod community in the central Baltic Sea (van Leeuwen et al. 2008).

Yodzis and Innes (1981, see also Calow and Townsend 1992) have argued that animal populations can be considered energy processors that transform energy from resources into new biomass and that organisms are often energy limited. If so, it is absolutely necessary for making reliable predictions regarding the dynamics of natural systems that a modeling framework accounts for incorporate size-dependent, individual-level energetics and food-dependence of energetic processes (Persson and de Roos 2013; Persson et al. 2014). The resulting intra-specific competition and density dependence of somatic growth and reproduction leads to surprising changes in population size distributions in response to mortality (de Roos et al. 2007; Schröder et al. 2009). Such changes may affect species at higher trophic levels because predation tends to be strongly size specific (Peters 1983; Ebenman and Persson 1988). Theory indicates that this mechanism can result in alternative stable states of a system with and without a predator present in the system (de Roos and Persson 2002; van Leeuwen et al. 2008). Empirical evidence of this type of alternative stable states stems from strongly seasonal systems (Persson et al. 2007). This calls for a robustness test of these phenomena to seasonal reproduction, which the model that we derived in this study allows for.

Species recruitment success is thought to be largely determined by the synchronicity of their reproductive peak with optimal food availability for their offspring (Cushing 1990; Durant et al. 2007). The effect of a match or mis-match between a species and its food source can permeate through several trophic levels in a system (Both et al. 2009; Nakazawa and Doi 2012; Revilla et al. 2014). Interactions between different species may change due to climatic changes. More specifically, the synchronicity between species over different ontogenetic life stages can make up species interactions and potentially determine a species success (Yang and Rudolf 2010; Rasmussen et al. 2014). For example, hatching phenology of predatory salamanders has been shown to determine their size-specific interaction with frog tadpoles (Nosaka et al. 2015). Hatching on time allows them to keep up with the growth of their prey and benefit more from predation. The modeling framework we present can be extended by adding other species (as explained above). In addition, it is possible to vary the timing of reproduction of the different species and investigate the effect of time lags between the reproduction of prey and predator.

The simplest way of representing differences between individuals in different life stages is with a stage-structured model with one juvenile, non-reproducing stage and one adult, reproducing stage. While this is an obvious simplification of the biological reality of complex life cycles, previous studies have shown that such a simple model already catches some essential characteristics of size-structured populations (Schröder et al. 2014). In addition, the two-stage model is a relatively small deviation from unstructured population models and therefore a suitable tool for comparisons with unstructured models and tests of established ecological theory. The multiple juvenile stages in the model we derived are energetically equivalent and have biologically no relevance besides how they change the recruitment rate of biomass to the adult stage. Differences between different life stages can easily be implemented by using stage-specific parameter values. In addition, it is possible to implement a diet- or niche-shift by adding resources and stage-specific diet preferences to the model (Guill 2009). The same applies for multiple adult stages, if the effect of growing adults is of interest (de Roos and Persson 2013).

The simultaneous maturation of all individuals in the cohort in the PSPM results from the assumption of deterministic growth and identical growth trajectories of all individuals in a cohort. In natural populations, maturation of a cohort of individuals born at the same time is probably more spread out over time than the delta function with which maturation is represented in the PSPM, due to individual variation in characteristics such as the size at birth and ingestion and metabolic rates. On the other hand, with the size-independent per-capita maturation rate for juveniles in the stage-structured biomass model, the moment of maturation varies widely between individuals. Reality lies probably somewhere in between these two extreme assumptions. In principle, this is approximated more faithfully by including multiple juvenile stages in our stage-structured biomass model.

An important dynamical consequence of the deterministic growth assumption in the PSPM is the occurrence of distinct and pronounced population cycles, in particular when juvenile consumers have a higher mass-specific ingestion rate than adults. These cycles are characterized by the dominance of the population by a single cohort of individuals and an alternation in time between juveniles and adults making up the largest part of the population. Such population cycles have theoretically been shown to affect coexistence possibilities of interacting species (Huss et al. 2013; van Leeuwen et al. 2014). Yet, an absolute separation in time between adults and juveniles within a population, which characterizes these types of cycles, is usually not observed in natural populations. In addition, simulations with individual-based models show that individual variation in the form of demographic stochasticity can break up these large amplitude population cycles (Nanthasubramaniam et al. 2011; Nisbet et al. 2016). Therefore, models that do not exhibit this extreme form of population cycles might provide a better baseline for studying population dynamics. Due to the spread out maturation over time, cycles occur less readily in the dynamics of the stage-structured biomass model, and, if they occur, have a smaller amplitude. An intermediate situation can be achieved by incorporating multiple juvenile stages in the stage-structured biomass model (de Roos et al. 2008; Guill 2009, results from this study). This decreases the probability of individuals maturing right after they are born and promotes the occurrence of population cycles.

Reiterating our main aim, we have in this paper derived a modeling framework that allows for the analysis of seasonally reproducing size-structured populations using one or multiple stages for juvenile and mature individuals. The relatively simple framework can easily be extended to ensembles of multiple interacting species. This creates the possibility to investigate the effect of seasonal reproduction on community dynamics while incorporating ontogenetic development and complex life histories in combination with seasonal reproduction.

## Appendix: Stage-structured biomass model with multiple juvenile stages

### Contribution of biomass to the adult stage

The expected lifetime contribution of biomass of a newborn individual to the adult stage is the same in the physiologically structured population model (PSPM) and the stage-structured biomass model. Yet, the dynamics over time of the biomass contribution to the adult stage differs between the two models. In this section, we compare the timing of the contribution of biomass of a newborn individual to the adult stage between the PSPM and stage-structured biomass model with one and multiple juvenile stages. In order to do this, we presume a situation with a constant resource density that is high enough to at least cover the maintenance costs. We suppress the resource dependent notation of the mortality, net-biomass production, and maturation terms into *ν*_*J*_, *μ*_*J*_ and *γ*, respectively.

The expected contribution of biomass of a newborn individual to the adult stage of a cohort in the PSPM follows a delta function over time. Maturation occurs when reaching size *Sm* that occurs at age $\frac {-\ln ({z} ) }{{ \nu _{J}}}$ (18). The contribution in terms of biomass to the adult stage by a single newborn individual thus equals:

$$Sm\,p(a_{m})= Sm\,{z}^{{\frac {\mu_{J}}{{ \nu_{J}}}}} = \frac{Sb}{z}\,{z}^{{\frac {\mu_{J}}{{ \nu_{J}}}}}=Sb\,{z}^{{\frac {\mu_{J}}{{ \nu_{J}}}}-1} $$

where *p*(*a*_*m*_) is the survival probability of an individual up to age *a*_*m*_ (19). The expected per-capita contribution of biomass to the adult stage over time *K*_*p*_ thus becomes:

33 $$ K_{p}(t)= \left\{\begin{array}{ll} 0 & \text{if} \quad t < \frac {-\ln ({z} ) }{{ \nu_{J}}}, \\ Sb \, {z}^{{\frac {\mu_{J}}{{ \nu_{J}}}}-1} & \text{if} \quad t > \frac {-\ln ({z} ) }{{ \nu_{J}}}, \end{array}\right. $$

for the PSPM.

In the stage-structured biomass model, we initially implement one juvenile stage *J*_1_. For a single new-born individual with body mass *Sb*, biomass in this stage changes over time as:

$$J_{1}(t) = Sb\,e^{(\nu_{J} - \mu_{J} - \gamma)\,t}\,. $$

And maturation of biomass over time follows:

34 $$ M_{1}(t) = \gamma\,Sb\,e^{(\nu_{J} - \mu_{J} - \gamma)\,t}\,. $$

The expected per-capita biomass contribution to the adult stage, *K*_*s*1_, as a function of time thus equals:

$$K_{s1}= {{\int}_{0}^{t}} \gamma\, Sb \, e^{(\nu_{J} - \mu_{J} - \gamma)\,x} \, \mathrm{d}x = \gamma\, Sb \, \frac{e^{(\nu_{J} - \mu_{J} - \gamma)\,t}-1}{\nu_{J} - \mu_{J} - \gamma}, $$

for a stage-structured biomass model with one juvenile stage. Filling in the maturation function (21) for *γ* results in:

35 $$ K_{s1}(t)= Sb \,(1-e^{\left( \frac{t\,(\nu_{J}-\mu_{J})}{1-z^{\frac{\mu_{J}}{\nu_{J}}-1}}\right)})z^{\frac{\mu_{J}}{\nu_{J}}-1}, $$

with only *z*, *ν*_*J*_ and *μ*_*J*_ as unknowns. Figure 1a in the main text compares *K*_*p*_(*t*) and *K*_*s*1_(*t*) for the default parameter values and the maximum value of *ν*_*J*_ = 0.015 for *q* = 1.

Adding a second juvenile stage *J*_2_ with identical energetics and parameters as the first juvenile stage to the stage-structured biomass model results in an ODE of the form:

$$\frac{dJ_{2}}{d\tau} = M_{1}(t) + (\nu_{J} - \mu_{J} - \gamma) J_{2}, $$

with general solution

$$J_{2}(t) = {{\int}_{0}^{t}} M_{1}(x) \, e^{(\nu_{J} - \mu_{J} - \gamma)\,(t-x)} \,\mathrm{d}x. $$

This describes the change of biomass in *J*_2_ over time when starting with one new-born individual at *t* = 0 (*J*_1_(0) = *Sb* and *J*_2_(0)=0). Substituting *M*_1_(*t*) from Eq. 34 results in:

$$\begin{array}{@{}rcl@{}} J_{2}(t) &=& {{\int}_{0}^{t}} \gamma\, Sb\, e^{(\nu_{J} - \mu_{J} - \gamma)\,x} \, e^{(\nu_{J} - \mu_{J} - \gamma)\,(t-x)} \,\mathrm{d}x \\&=& t\,\gamma \, Sb \, e^{(\nu_{J} - \mu_{J} - \gamma)\,t}. \end{array} $$

So, maturation of biomass at time *t* out of the second juvenile stage equals:

$$M_{2} (t)= t\,\gamma^{2} \, Sb \, e^{(\nu_{J} - \mu_{J} - \gamma)\,t}. $$

More generally, with *n* identical juvenile stages the maturation rate at time *t* out of the *n*th juvenile stage is given by:

36 $$ M_{n}(t) = \frac{1}{(n-1)!}\,t^{(n-1)}\,\gamma^{n}\,Sb\, e^{(\nu_{J}-\mu_{J}-\gamma)\,t}\,. $$

Resulting in the following general formula for *K*_*sn*_, the expected per-capita biomass contribution to the adult stage over time:

37 $$\begin{array}{@{}rcl@{}} K_{sn}& =\, & Sb \,{{\int}_{0}^{t}} \frac{1}{(n-1)!}\,x^{(n-1)}\,\gamma^{n}\, e^{(\nu_{J}-\mu_{J}-\gamma)\,x} \, \mathrm{d}x\\ \\ &=\, & Sb\, t^{n}\,\gamma^{n}\, e^{(\nu_{J}-\mu_{J}-\gamma)\frac{1}{2}\,t}\,\big((\nu_{J}-\mu_{J}-\gamma)\,-t\big)^{-\frac{1}{2}n} \\ \\ &&\times\left( \frac{\text{WhittakerM}\big(\frac{1}{2}\,n,\frac{1}{2}\,n+\frac{1}{2},((\nu_{J}-\mu_{J}-\gamma)\,-t)\big)}{(n+1)!}\right. \\ \\ &&-\left.\frac{\text{WhittakerM}\big(\frac{1}{2}\,n + 1,\frac{1}{2}\,n+\frac{1}{2},((\nu_{J}-\mu_{J}-\gamma)\,-t)\big)}{(\nu_{J}-\mu_{J}-\gamma)\,t\,n!} \right). \\ \end{array} $$

Parameters and functions are all as described in Section 1 in the main text of this manuscript except for maturation function *γ* that in case of multiple juvenile stages is described by:

38 $$ \gamma (\nu_{J}(R)) = \left\{\begin{array}{ll} \displaystyle\frac { \nu_{J} (R) - \mu_{J}} {1-(z_{n})^{1-\frac{\mu_{J}}{\nu_{J}(R)}}}, & \text{if} \quad \nu_{J} (R) > 0, \\ 0, & \text{otherwise}, \end{array}\right. $$

where we replaced *z* by *z*_*n*_ compared to Eq. 22. If we assume all juvenile stages to enclose the same scope of growth between *Sb* and *Sm* (*z* = *Sb*/*Sm*), *z*_*n*_ should be replaced in Eq. 38 by *z* ^1/*n*^. Given the resulting expression for *γ*, the expression (37) for *K*_*sn*_ only contains *z*, *ν*_*J*_, and *μ*_*J*_ as unknowns. From these, *z* and *μ*_*J*_ are model parameters given in Table 1. The curves shown in Fig. 1b are based on a situation where *n* = 2,3, or 4 and *ν*_*J*_ = 0.015, its maximum value for *q* = 1.

### System of ODEs for stage-structured biomass model with three juvenile stages

The full system of ordinary differential equations for a stage-structured biomass model with three juvenile stages of equal size range as a function of time for the dynamics in the interval between reproductive events, *Y*, (0≤*τ*<*Y*) is:

39 $$\begin{array}{@{}rcl@{}} \frac{dJ_{1}}{dt}& =& (\nu^{+}_{J}(R) - \gamma (\nu_{J}(R)) - d_{J}(R) ) \,J_{1} \end{array} $$

40 $$\begin{array}{@{}rcl@{}} \frac{dJ_{2}}{dt}& =& \gamma (\nu_{J}(R)) \, J_{1} \\&&+ (\nu^{+}_{J}(R) - \gamma (\nu_{J}(R)) - d_{J}(R) ) \,J_{2} \end{array} $$

41 $$\begin{array}{@{}rcl@{}} \frac{dJ_{3}}{dt}& =& \gamma (\nu_{J}(R)) \, J_{2} \\&&+ (\nu^{+}_{J}(R) - \gamma (\nu_{J}(R)) - d_{J}(R) ) \, J_{3} \end{array} $$

42 $$\begin{array}{@{}rcl@{}} \frac{dA}{dt} & =& \gamma \, (\nu_{J}(R)) J_{3} - d_{A}(R) A \end{array} $$

43 $$\begin{array}{@{}rcl@{}} \frac{dB}{dt} &=& {\nu^{+}_{A} (R)} {A} - d_{A}(R) {B} \end{array} $$

44 $$\begin{array}{@{}rcl@{}} \frac{dR}{dt} & =& \delta \, (R_{max} - R) - ((2-q) (J_{1}+J_{2}+J_{3}) + q\,A) \\&&\times M \frac{R}{H + R}\,. \end{array} $$

Parameters and functions are all as described in Section 1 in the main text of this manuscript except for the maturation function *γ* that is now described by Eq. 38. In this latter function, we replaced *z*_*n*_ by *z* ^1/3^, as we assume all juvenile stages to enclose the same scope of growth between *Sb* and *Sm*, where *z* = *Sb*/*Sm*. Seasonal reproduction takes place instantaneously at intervals *t*_*n*_ = *nY*:

45 $$\begin{array}{@{}rcl@{}} J_{1}(t_{n}^{+})&=&J_{1}(t_{n}^{-}) + B(t_{n}^{-}) \end{array} $$

46 $$\begin{array}{@{}rcl@{}} J_{2}(t_{n}^{+})&=&J_{2}(t_{n}^{-}) \end{array} $$

47 $$\begin{array}{@{}rcl@{}} J_{3}(t_{n}^{+})&=&J_{3}(t_{n}^{-}) \end{array} $$

48 $$\begin{array}{@{}rcl@{}} A(t_{n}^{+})&=&A(t_{n}^{-}) \end{array} $$

49 $$\begin{array}{@{}rcl@{}} B(t_{n}^{+})&=&0 \end{array} $$

50 $$\begin{array}{@{}rcl@{}} R(t_{n}^{+})&=&R(t_{n}^{-})\,. \end{array} $$

At a reproductive event, the biomass of the first juvenile stage increases with the biomass in the reproductive storage (45) and the biomass of the storage is set to 0 (49). The other stages of the consumer population do not change.
